# Applying oversampling before cross-validation will lead to high bias in radiomics

**DOI:** 10.1038/s41598-024-62585-z

**Published:** 2024-05-21

**Authors:** Aydin Demircioğlu

**Affiliations:** Institute of Diagnostic and Interventional Radiology and Neuroradiology, University Hospital Essen, Hufelandstraße 55, 45147 Essen, Germany

**Keywords:** Predictive markers, Data processing, Image processing, Machine learning, Statistical methods

## Abstract

Class imbalance is often unavoidable for radiomic data collected from clinical routine. It can create problems during classifier training since the majority class could dominate the minority class. Consequently, resampling methods like oversampling or undersampling are applied to the data to class-balance the data. However, the resampling must not be applied upfront to all data because it would lead to data leakage and, therefore, to erroneous results. This study aims to measure the extent of this bias. Five-fold cross-validation with 30 repeats was performed using a set of 15 radiomic datasets to train predictive models. The training involved two scenarios: first, the models were trained correctly by applying the resampling methods during the cross-validation. Second, the models were trained incorrectly by performing the resampling on all the data before cross-validation. The bias was defined empirically as the difference between the best-performing models in both scenarios in terms of area under the receiver operating characteristic curve (AUC), sensitivity, specificity, balanced accuracy, and the Brier score. In addition, a simulation study was performed on a randomly generated dataset for verification. The results demonstrated that incorrectly applying the oversampling methods to all data resulted in a large positive bias (up to 0.34 in AUC, 0.33 in sensitivity, 0.31 in specificity, and 0.37 in balanced accuracy). The bias depended on the data balance, and approximately an increase of 0.10 in the AUC was observed for each increase in imbalance. The models also showed a bias in calibration measured using the Brier score, which differed by up to −0.18 between the correctly and incorrectly trained models. The undersampling methods were not affected significantly by bias. These results emphasize that any resampling method should be applied correctly only to the training data to avoid data leakage and, subsequently, biased model performance and calibration.

## Introduction

Radiomics has recently emerged as a key method in analyzing radiological imaging data^1,2^. It can be used for various tasks, including the quantification of imaging^3^, the characterization of tumors^4^, and for diagnosis and prognosis^5,6^. Radiomics can be understood as the application of a machine learning pipeline to radiological data obtained from clinical routine^7,8^. However, the radiological data may be class-imbalanced, meaning that one class is more prevalent in the data than others, due to small sample sizes or, occasionally, due to rare diseases. In such scenarios, the training of a classifier might be challenging since the majority class could potentially dominate the predictions, indicating that the classifier is biased towards the majority class.

To avoid this situation, resampling methods are often employed to class-balance the data. These methods can be divided primarily into three classes: (1) oversampling methods, which generate new samples for the minority class; (2) undersampling methods, which discard samples of the majority class; and (3) combined methods, which apply both techniques to obtain a balanced dataset.

However, these methods must be applied in a methodologically correct manner to avoid obtaining erroneous results and, consequently, false positive results^9^. An issue that often arises in machine learning is data leakage, which occurs when the data on which the final model is tested has also been used during training. This breaks one of the golden rules of machine learning, which states that all modeling must be performed only on the training data^10,11^. If the test data has already been seen during training, the model’s performance on the test data would be biased and could lead to false positive findings. Here, we define bias empirically as the difference in a metric between a model that has no apparent data leakage and a model that exhibits such leakage when both are evaluated on an independent data set.

**Figure 1:**
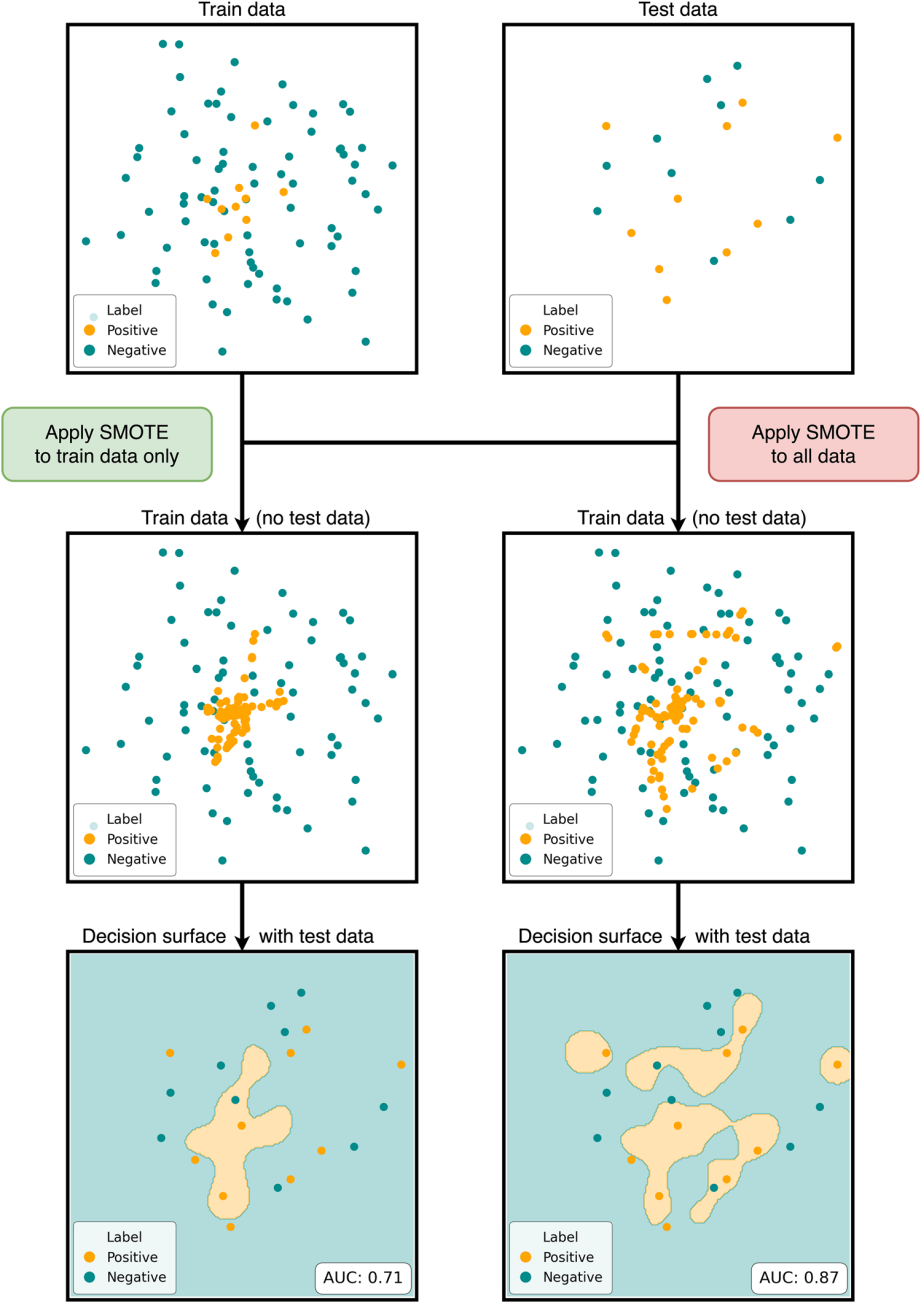
Illustration of the bias when the resampling is misapplied. Left side: correct application, leading to an AUC of 0.71. Right side: incorrect application, leading to a biased AUC of 0.87. Note that the training data on the both sides contain no test samples; however, the on the right side the oversampling created samples very close to samples in the test set, which is the reason for the data leakage.

The application of any resampling methods must also follow this rule: they must not be applied to all data upfront but only to the training data. To illustrate this issue, consider the synthetic data represented in Fig. 1. Applying an oversampling method—in this case, the often-used Synthetic Minority Oversampling Technique (SMOTE)—to all data rather than solely to the training data can lead to a biased classifier that can predict small areas in the test set where the prediction is positive, although the training data did not contain related information. In this example, an increase of + 0.16 is observed in the area under the receiver operating characteristic curve (AUC), which can be attributed purely to the data leakage since every other parameter was fixed.

Although data leakage should always be avoided, this type of misapplication of resampling methods has seemingly been performed in a few radiomics studies^12–14^. Consequently, the question of the degree of bias arises, resulting in uncertainty regarding whether the results of such studies can be trusted.

To estimate how large the bias could be if resampling techniques are applied incorrectly in the radiomics domain, several resampling methods are applied in this paper, including over- and undersampling, once correctly and once incorrectly. Subsequently, the empirical bias, that is, the difference in terms of predictive performance and model calibration, is measured using the AUC and Brier score. In addition, the bias in sensitivity, specificity, and balanced accuracy is determined. Finally, a simulation study is conducted to analyze the bias on a randomly generated synthetical dataset.

## Results

### Observed bias in predictive performance

**Figure 2:**
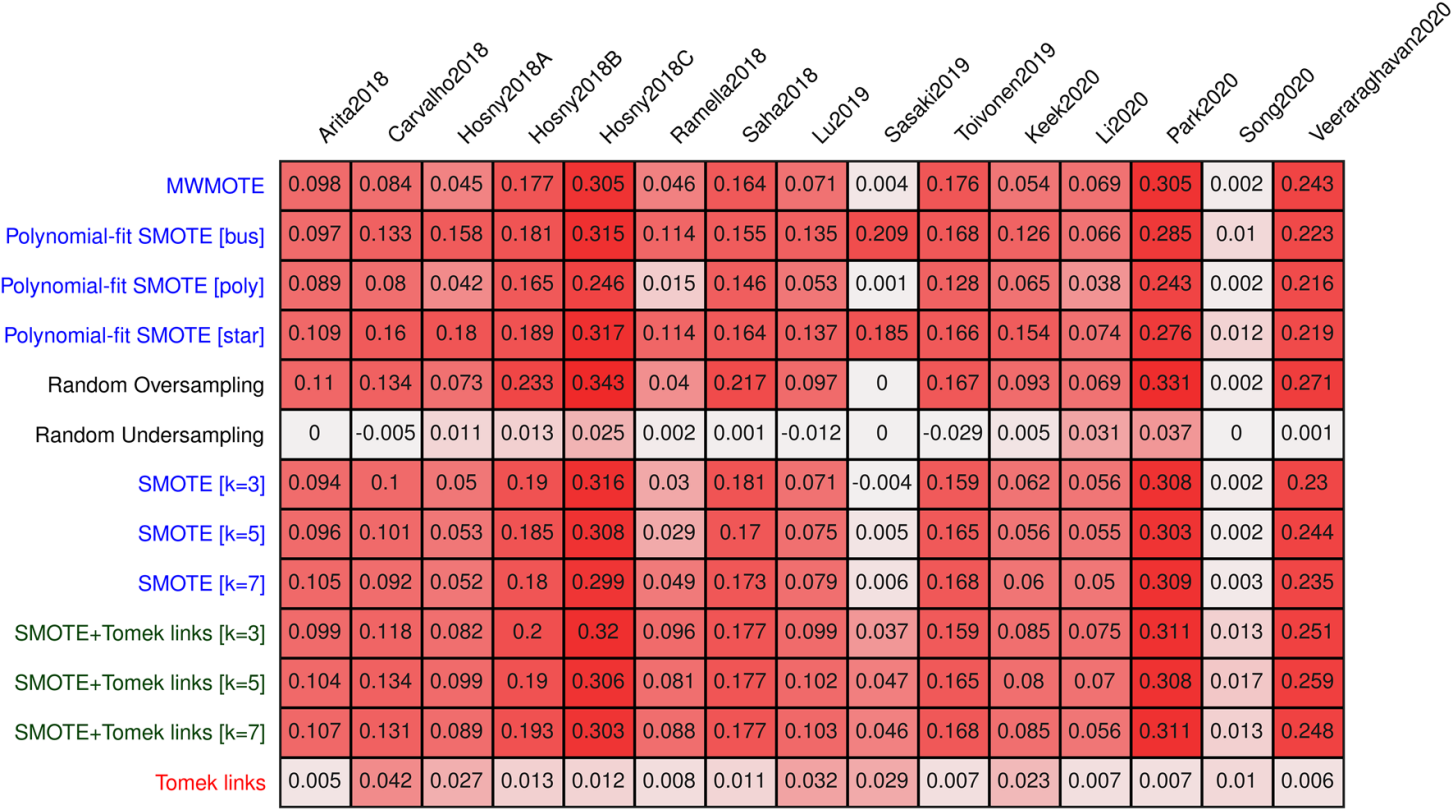
Bias in AUC of the best-performing models averaged over 30 repeats for each method and dataset. Undersampling methods are displayed in red, combined in green and oversampling methods in red.

**Figure 3:**
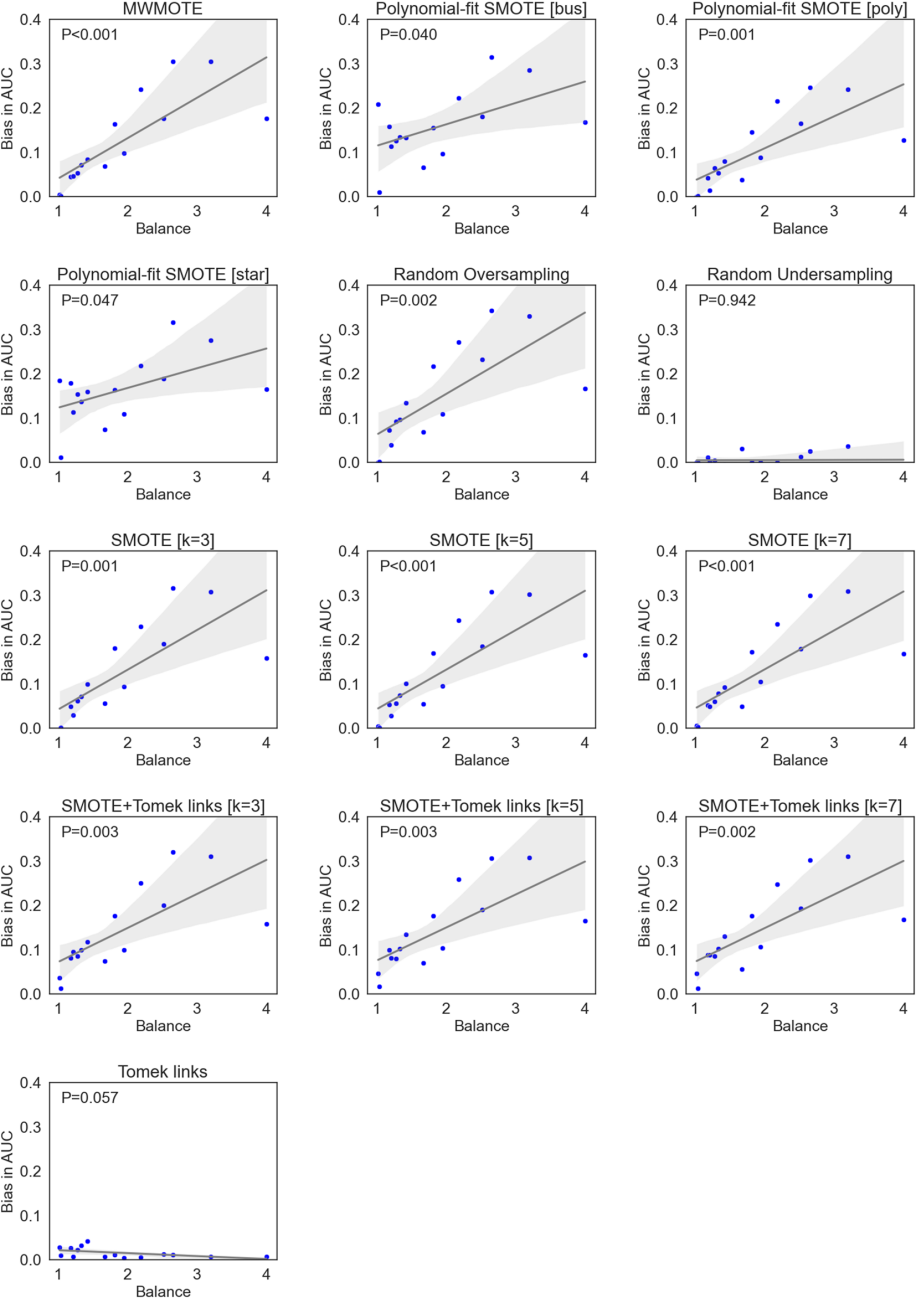
Association of the bias in AUC with the class-balance of each dataset. The grey area denotes the 95% confidence interval.

The incorrect application of the selected resampling methods led to a high bias in AUC for all methods except for the two undersampling methods (Fig. 2). Notably, the undersampling methods were less affected than the combined or oversampling methods (up to 0.042 in AUC for the undersampling methods vs. 0.343 for the latter). A highly significant association between the observed bias and the balance of the data was observed for all methods except the undersampling methods (Fig. 3). A similar pattern was observed for sensitivity (up to 0.33, Supplementary Materials S1), specificity (up to 0.37, Supplementary Materials S1), and balanced accuracy (up to 0.31, Supplementary Materials S1).

### Observed bias in Brier score

**Figure 4:**
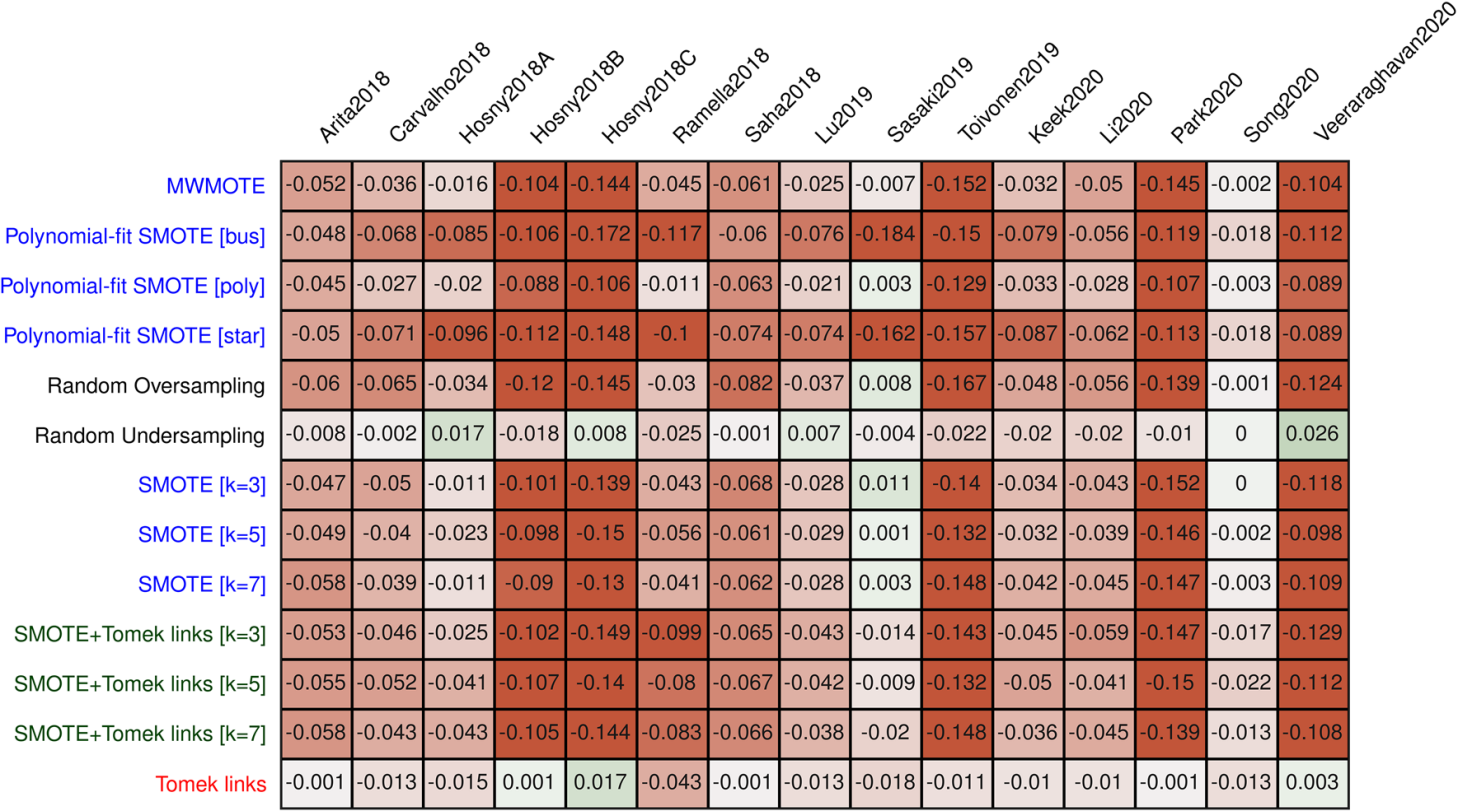
Bias in Brier score of the best-performing models averaged over 30 repeats for each method and dataset. Undersampling methods are displayed in red, combined in green, and oversampling methods in red.

**Figure 5:**
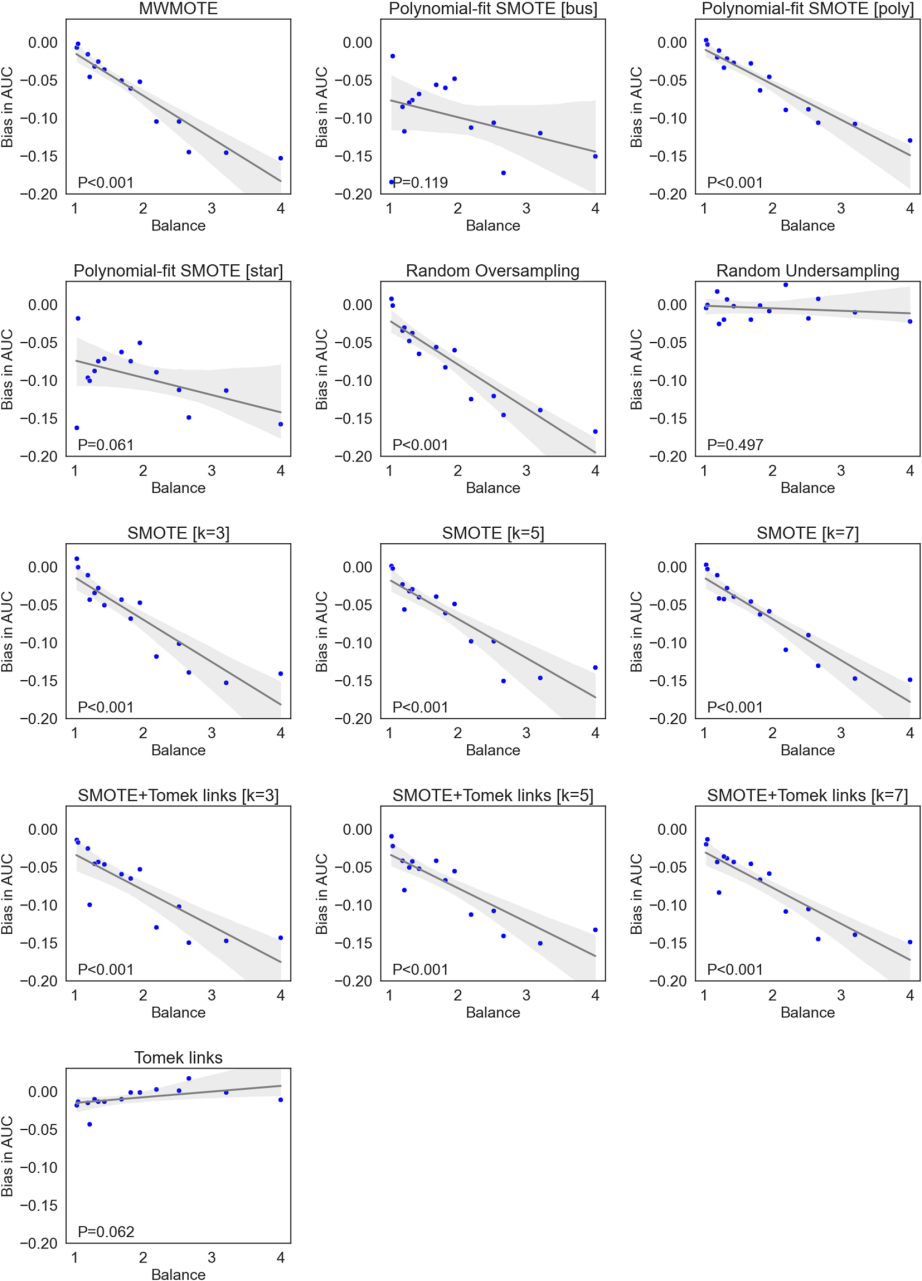
Association of the bias in Brier score with the class-balance of each dataset. The grey area denotes the 95% confidence interval.

Similarly, a bias was observed in the model calibration, measured using the Brier score (Fig. 4). The application of the oversampling and combined methods led to an improved Brier score, while the undersampling methods were affected by this bias only marginally. Notably, undersampling methods were less affected than the combined or oversampling methods, with a bias of up to −0.043 and −0.184, respectively. A highly significant association between the observed bias and the balance of the data was observed for all methods except the undersampling methods (Fig. 5).

### Simulation study

**Figure 6:**
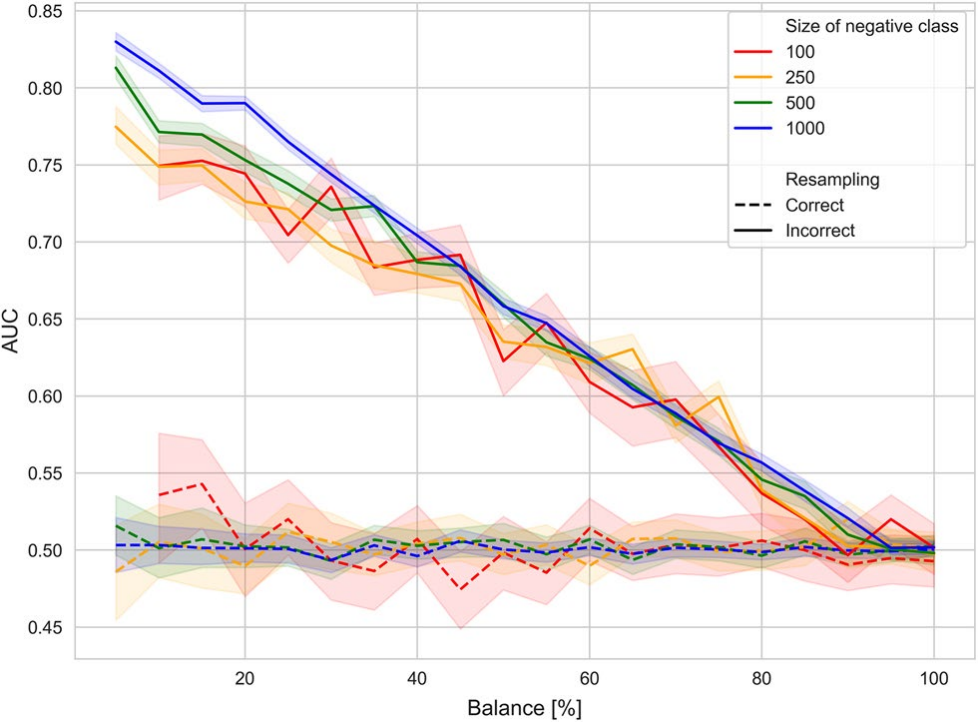
Results of the simulation study. Different sample sizes were used to determine its effect on the observed bias. 95% confidence intervals are drawn shaded.

In the simulation study, a clear difference between the results when SMOTE was applied correctly and incorrectly was observed, too (Fig. 6). Since all the data were sampled randomly, no classifier was expected to perform better than a random classifier. Indeed, the correct application produced classifiers that achieved an AUC of approximately 0.5. In stark contrast and largely independent of the overall sample size, the incorrect application of SMOTE led to models that performed much better than random and can, therefore, be identified as biased. A relatively linear association between the balance in the dataset and the bias was also observed, with an approximate increase of 0.10 in the AUC per increase in imbalance.

## Discussion

Radiomic datasets contain some class imbalance, which is often unavoidable in data gathered from clinical routine. Resampling the data during training to achieve class balance can be important to obtain properly calibrated and well-performing models. However, to avoid data leakage the resampling must be performed only on the training data. In our study, we analyzed the degree of bias introduced if resampling was wrongly applied to all the data rather than only the training data, and observed a high bias in both predictive performance and model calibration.

Our results reveal that the misapplication of resampling techniques leads to a very high bias, especially for oversampling and combined methods. The observed bias in performance reached up to 0.34 in AUC. A similar bias was also observed in sensitivity, specificity, and balanced accuracy. While the degree of this bias in predictive performance differed across the datasets used, its association with the balance of the data was evident: nearly balanced datasets led to smaller amounts of bias compared to datasets with greater imbalance. However, no significant bias was observed when applying the two undersampling methods, namely, random undersampling and Tomek links.

This behavior is not unexpected since, when resampling techniques are misapplied, every generated synthetical sample potentially carries some information on the test set. Consequently, the more imbalanced a dataset was, the more such samples were generated. This association was significant for all methods that generated samples synthetically. However, the otherwise high-performing methods based on polynomial-fit SMOTE using bus and star topology exhibited a high bias, even in the case of balanced datasets^15,16^. It could be because both methods, in contrast to the other methods, always generate synthetical samples, regardless of the balance of the underlying dataset.

Similar results were observed for the calibration of the resulting models. Applying the balancing upfront led to a high bias in the Brier score (up to -0.14) for the oversampling and combined methods while no clear bias was observed for the undersampling methods. Again, the relationship depended very strongly on the balance of the dataset; imbalanced data, where many synthetical samples were generated, exhibited much higher bias.

While these are more empirical results that depend on various factors, such as the feature selection method and the classifier used, we also performed a simple simulation using data with random outcomes, on which no model can perform better than chance. Yet, as expected, the models that were trained incorrectly performed much better than the correctly trained models, achieving an AUC of up to 0.90 during testing. This result highlights our empirical findings based on the different datasets: each increase in imbalance yields an approximate increase of 0.1 in the AUC. Since the value of the AUC cannot be larger than 1.0, this effect eventually reaches saturation.

The misapplication of resampling methods and the consequent bias in the results of studies in which the data leakage occurred often cannot be observed directly. Moreover, it must be noted that many radiomics studies lack adequate descriptions of their methodology that may allow them to be fully reproducible^17^. However, the misapplication of resampling techniques would be noticed if the model was tested on other data, but such independent or external testing is often neglected in many studies due to the increased efforts that may be required^17^. If such misapplication occurred and was not noticed, it is reasonable that the results based on the test data are much lower than previously seen and expected^18^. It is likely that such studies would blame an overfitting of the classifier or the low reproducibility and instability of the radiomic features for the inferior results.

Nonetheless, we estimate that the misapplication of resampling techniques is not widespread. To gauge this, we reviewed papers published in 2023 using the keywords ‘SMOTE’, ‘oversampling’, ‘undersampling’, ‘imbalance’ and ‘radiomics’ (see Supplementary Materials S1 and Supplementary Table S2). We identified five among a total of 34 papers (5/34, 15%) in which the reporting highly suggests that misapplication occurred. The reporting of the used methods was vague in another study, from which no conclusion could be drawn. Among the studies in which we assumed a misapplication or could not determine its occurence, only one shared their data^19^; however, the data does not contain the relevant outcomes, rendering it useless for predictive modeling.

To illustrate the degree of bias, we consider a proof-of-concept study by Hinzpeter et al., who claim that bone metastases from prostate cancer can be detected in CT imaging using radiomics even though they cannot be seen by the naked eye^13^. They state that their method achieved an AUC (incorrectly referred to as accuracy) of 0.85. Their analysis, however, describes that the oversampling method, Majority Weighted Minority Oversampling (MWMOTE), was applied before splitting the data into training and test sets. Since the balance of their data is roughly 2.3, assuming that a linear relationship exists between balance and bias, we estimate that this study shows a bias of approximately 0.15–0.20 in the AUC. Thus, the true, unbiased AUC could be close to 0.65–0.70. This value questions their claim that bone metastases invisible to radiologists can be detected with high accuracy using radiomics.

Another interesting example is the study by Liu et al., which proposes a model to predict lung metastases in patients with thyroid cancer^20^. Their data is extremely imbalanced since only 212 out of 9738 patients had lung metastases. They also incorrectly perform resampling before training but test the over- and undersampling methods. Accordingly, they report that the oversampling method performed better, with a difference of approximately 0.14 in the AUC for the two methods (0.99 for oversampling and 0.85 for undersampling). Assuming that undersampling is relatively unbiased, this difference can be understood as a positive bias and indicates that the prediction of lung metastases is good but not excellent, as suggested in the study. Our rule of thumb—for each imbalance of the data, a bias of 0.10 can be expected—appears to be violated here; however, this is because the AUC of the model is already near perfect and cannot improve beyond 1.0.

A limited number of studies specifically consider this problem. A recent study by Vandewiele et al. considers the bias of oversampling methods in a single dataset, the Term-Preterm EHG Database, which is a collection of electrohysterogram recordings and has an imbalance of approximately 1:7^21^. They report a bias in the range of 3–10%, which is lower than what we observed in our study; however, it is important to keep in mind that their preprocessed dataset has fewer features (d = 50) than samples (N = 398) and is not directly comparable to a radiomics dataset. However, they conduct a simulation study using five-dimensional synthetically generated data with an imbalance of 1:10 and report a bias of + 0.45 in the AUC, which is higher than the results we achieved in our simulation study.

We reiterate that the results of our study are not surprising. Using the test data in any way during training is a form of data leakage and will lead to a bias in the majority of cases. Even though the application of undersampling methods appears to produce relatively unbiased results, applying them upfront to all data is still incorrect and should be avoided. The misapplication of the resampling methods is only one source of data leakage, which can occur on several levels and yield a large positive bias^9,22–25^. For example, while Kawahara et al. apply resampling techniques correctly, they divide the train and test sets only after applying the least absolute shrinkage and selection operator (LASSO)^26^. Therefore, their results could potentially be biased^22^.

It should be noted that our study has certain limitations. We only considered data with binary outcomes. However, it is reasonable to expect that similar biases will also occur for other types of outcomes since the underlying problem, data leakage, will also be present in such scenarios. Although our results indicate that the misapplication of oversampling methods leads to bias, this bias may still depend strongly on the dataset. We also only consider five-fold cross-validation. Nevertheless, we expect similar results to hold for other validation schemes as well, as long as data leakage occurs. We cannot exclude other forms of bias or data leakage in the datasets or the methods we have employed. For example, we normalize all data upfront, which results in another form of data leakage. However, a recent study suggests that this method is relatively unbiased^27^. Moreover, it would affect all methods similarly. Furthermore, although various resampling methods exist^15^, only a few that are widely used in the radiomics domain are considered in this study. We cannot conclude that other oversampling methods are also biased or that undersampling methods are inherently unbiased. The same is true for the metrics used to measure bias: while we employ often-used metrics, we are confident that similar biases exist even if other metrics are used, such as the F1 score, area under the precision-recall curve (AUPRC), and similar. Finally, some resampling algorithms have hyperparameters that need to be tuned; for example, in the case of SMOTE, the relevant hyperparameter is a simple k value that determines the number of neighbors to consider. More complex methods like MWMOTE may have several hyperparameters that require tuning. While we do not consider the broader implications of these hyperparameters, except in a simple case, tuning them using all the data, as often happens in case of misapplication of resampling techniques, is likely to increase the bias. Thus, our results can be considered a lower bound on the expected bias in any scenario.

### Conclusion

Incorrectly applying resampling methods to all data can lead to high bias in terms of model performance and model calibration in radiomics.

## Methods

### Ethical statement

In this study, we gathered several publicly available datasets; the corresponding approvals from the ethical review boards have been granted. Ethical approval for this study was waived by the local Ethics Committee (Ethik-Kommission, Medizinische Fakultät der Universität Duisburg-Essen, Germany). All methods and procedures were performed following the relevant guidelines and regulations. This is a retrospective study using only previously published and publicly accessible datasets. Approval was granted due to its retrospective nature.

### Datasets

Fifteen publicly available radiomic datasets in tabular form were collected unsystematically (Table 1). All datasets were preprocessed as follows: non-radiomic features, such as clinical or genetic features, were removed. Missing values were imputed with column means. All data were normalized using the z-score method. If a train/test split was available, it was discarded by merging all data splits. Furthermore, if the outcome was not binary, e.g., in the case of survival, it was dichotomized.

**Table 1:** Datasets used in this study.

Dataset	N	d	N+	N−	Balance	Modality	Tumor type	DOI
Arita2018	168	685	111	57	1.95	MRI	Brain	10.1038/s41598-018-30273-4
Carvalho2018	262	118	154	108	1.43	FDG + PET	NSCLC	10.1371/journal.pone.0192859
Hosny2018A	293	985	159	134	1.19	CT	NSCLC	10.1371/journal.pmed.1002711
Hosny2018B	211	1005	60	151	2.52	CT	NSCLC	10.1371/journal.pmed.1002711
Hosny2018C	183	1005	133	50	2.66	CT	NSCLC	10.1371/journal.pmed.1002711
Ramella2018	91	243	50	41	1.22	PET + CT	NSCLC	10.1371/journal.pone.0207455
Saha2018	922	530	327	595	1.82	DCE-MRI	Breast	10.1038/s41416-018-0185-8
Lu2019	213	658	91	122	1.34	CT	Ovarian cancer	10.1038/s41467-019-08718-9
Sasaki2019	138	588	68	70	1.03	MRI	Brain	10.1038/s41598-019-50849-y
Toivonen2019	100	7106	80	20	4	MRI	Prostate cancer	10.1371/journal.pone.0217702
Keek2020	273	1323	119	154	1.29	CT	HNSCC	10.1371/journal.pone.0232639
Li2020	51	397	32	19	1.68	MRI	Glioma	10.1371/journal.pone.0227703
Park2020	768	941	183	585	3.2	US	Thyroid cancer	10.1371/journal.pone.0227315
Song2020	260	265	127	133	1.05	MRI	Prostate cancer	10.1371/journal.pone.0237587
Veeraraghavan2020	150	201	47	103	2.19	DCE-MRI	Breast	10.1038/s41598-020-72475-9

N denotes the sample size, d the dimension (number of features). N+ denotes the number of samples with positive outcome, N− those with negative outcome. Balance is computed as the sample size of the majority class divided by the sample size of the minority class.

### Resampling methods

All three types of resampling methods were considered (Table 2). They included four oversampling methods (random oversampling, SMOTE, polynomial-fit SMOTE, and MWMOTE), two undersampling methods (random undersampling, Tomek links), and one combined method (SMOTE + Tomek links). These methods were selected empirically because they are either employed in radiomics often or performed very well in previous studies^15^. The default values of the hyperparameters of these methods were retained, except for SMOTE and SMOTE + Tomek links, for which *k* was varied between 3, 5, and 7, and for polynomial-fit SMOTE, where the topology was varied between star, bus and polynomial.

**Table 2:** List of resampling methods and parameters.

Method	Parameters	Type
Random undersampling	–	Undersampling
Tomek links	–	Undersampling
Random oversampling	–	Oversampling
SMOTE	k = 3, 5, 7	Oversampling
Polynomial-fit SMOTE	Topology = bus, star, poly	Oversampling
MWMOTE	–	Oversampling
SMOTE + Tomek links	k = 3, 5, 7	Combined

### Model training

Models were trained using the standard radiomics pipeline, consisting of a feature selection method and a classifier^28,29^. For the selection of relevant features, four commonly used feature selection methods were used^30^: analysis of variance (ANOVA), Bhattacharyya scores, extra trees (ET), and the least absolute shrinkage and selection operator (LASSO). Using these methods, each feature is scored according to its estimated relevance; however, it is a priori unclear how many features should be used. Therefore, this number was considered a hyperparameter, and different numbers of highest-scoring features were selected (N = 1, 2, 4, … 32, 64). A classifier was then trained based on these features. Four widely used classifiers were employed^31^: logistic regression (LR), Naive Bayes, random forest (RF), and kernelized support vector machines (RBF-SVM). The hyperparameters for these methods were tested using a grid-like scheme (Table 3).

**Table 3:** List of resampling methods and parameters.

	Method	Parameters
Feature selection	LASSO	C = 1.0
	Extra trees	–
	ANOVA	–
	Bhattacharyya	–
Classifiers	Logistic regression	C = 2^–10^, 2^–9^, …, 2^9^, 2^10^
	Naive Bayes	–
	RBF-SVM	C = 2^–10^, 2^–9^, …, 2^9^, 2^10^ , γ = auto
	Random forest	N = 250
	k-NN	k = 1, 3, 5, 7, 9

**Figure 7:**
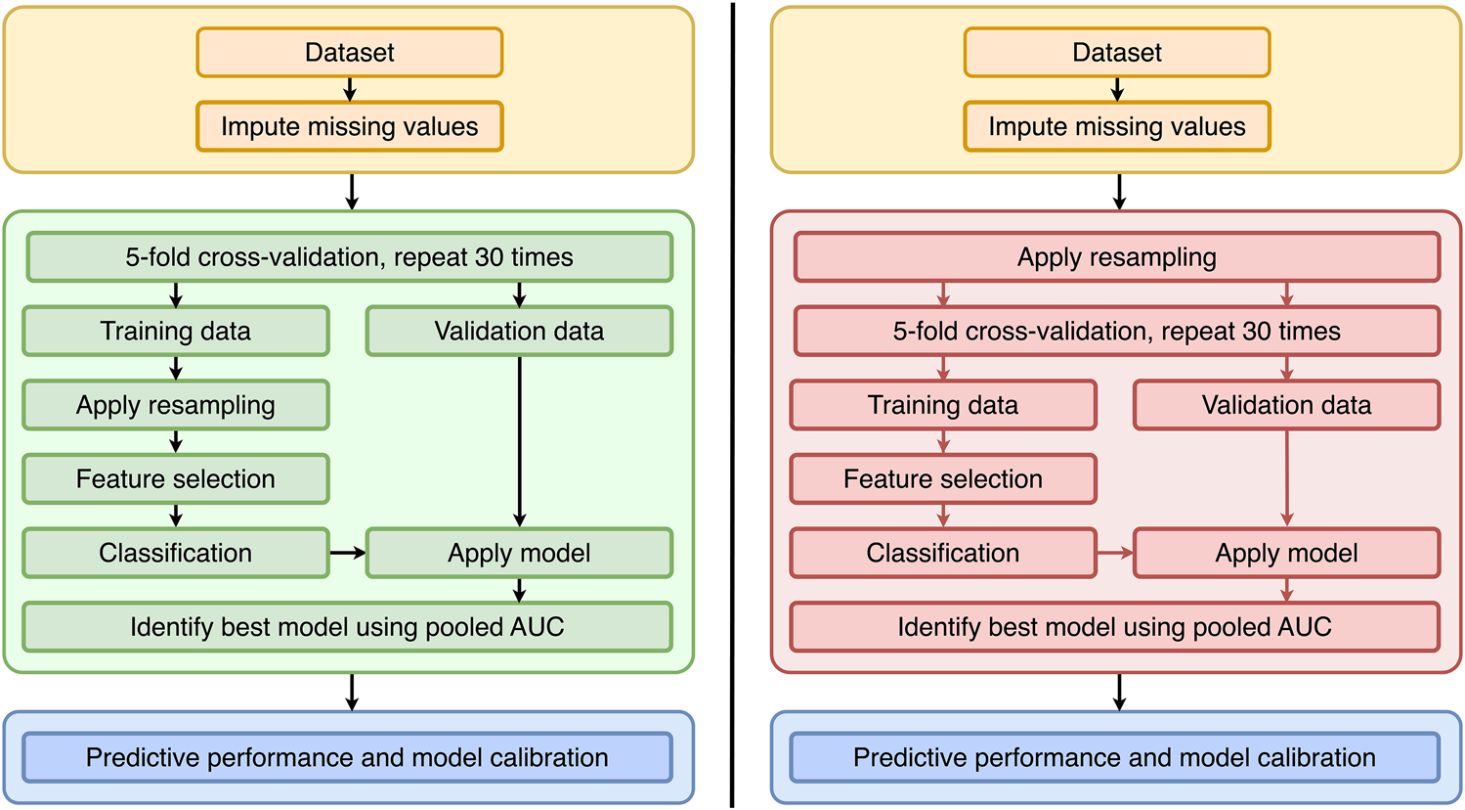
Flowchart of the two methodologies. On the left, the resampling is applied correctly within the cross-validation folds, on the right it is applied incorrectly before the cross-validation.

The training was performed using a fivefold stratified cross-validation (CV) with 30 repeats. Two different strategies were used (Fig. 7). First, the resampling was performed correctly during the CV, i.e., after splitting the data into train and test folds. In this scheme, the resampling was only applied to the training data, and the test data was not affected by the resampling. Second, the resampling was performed incorrectly before the CV. In this scenario, all the data were resampled before splitting. Therefore, the test data during the CV was affected by the resampling.

The best-performing model in each repeat in terms of the macro-averaged area under the receiver operator characteristic curve (AUC) was determined in both strategies. This model was then used to compute the bias.

### Observed bias in predictive performance

Since the primary focus in radiomics is obtaining accurate predictions, the difference in the AUC of the best-performing models for each scenario was computed. The AUC values across the repeats were then averaged. A scatter plot depicting the balance against the mean bias for each dataset was generated to determine whether the observed bias depends on the number of synthetically created points.

Similarly, the biases in the sensitivity, specificity, and the balanced accuracy were computed as secondary metrics.

### Observed bias in Brier score

Calibration bias was measured using the mean difference in the Brier score between the best-performing models across repeats. Similarly, a scatter plot was used to depict the association between the balance and the observed mean bias in the Brier score.

### Simulation study

A simple simulation study was performed to estimate the bias. Two-dimensional synthetic data was sampled uniformly from the range [−1, 1] with a pre-specified balance B (in %) and N negative samples. First, N samples were drawn randomly and assigned a negative label. Then, N*B/100 samples were drawn and assigned a positive label. Then, the data was processed, once correctly and once incorrectly, using SMOTE with the default neighborhood size (k = 5), where a repeated 80–20% train-test split with 100 repeats was used instead of a repeated CV. An SVM with an RBF kernel was used as a classifier. Large values for the parameters C and gamma were chosen (C = 50, gamma = 500) to increase the complexity of the classifier, which in turn determines whether the classifier can fit the data with high accuracy. The difference in AUC between both approaches was then plotted against the balance of the data.

### Software

All modeling was performed using Python 3.9 and the scikit-learn package. Our code repository can be found on GitHub (https://github.com/aydindemircioglu/ResamplingBias) and was archived on Zenodo (10.5281/zenodo.10890212).

### Ethic approval and consent to participate

This is a retrospective study using only previously published and publicly accessible datasets. Written informed consent was waived by the local Ethics Committee (Ethik-Kommission, Medizinische Fakultät der Universität Duisburg-Essen, Germany). Approval was granted due to its retrospective nature. All methods and procedures were performed following the relevant guidelines and regulations.

### Supplementary Information

Supplementary Information.

Supplementary Table S2.

## Data Availability

All datasets are publicly available. Code, data and results can be found on the public repository at https://www.github.com/aydindemircioglu/ResamplingBias.
