# Supplementary material for: Applying oversampling before cross-validation will lead to high bias in radiomics
Source: Sci Rep. 2024 May 21;14:11563. doi: 10.1038/s41598-024-62585-z (PMC11109211; doi:10.1038/s41598-024-62585-z)
Supplement: Supplementary file 1 — Supplementary Information. [file 41598_2024_62585_MOESM1_ESM.docx]

**Applying resampling before cross-validation will lead to high bias in radiomics**

**SUPPLEMENTARY MATERIAL**

**S1. Review of papers in 2023 that apply resampling**

We performed a small review of papers published in 2023 to estimate how many published studies could be affected by misapplication of resampling methods. We queried Pubmed and Scopus using the following four keyword combinations: ‘oversampling AND radiomics’, ‘undersampling AND radiomics’, ‘resampling AND radiomics’, ‘SMOTE AND radiomics’. Each study was then checked whether the resampling was reported to be performed on the training set only. However, since only very few of the studies published their code, the judgment can only be done by the reporting in the paper. This might be inaccurate, since the description might be either deliberately brief to avoid a too technical language when the paper is geared towards non-machine learning experts, or in some cases the relevant section might be written by a non-expert, although the analysis itself was performed correctly. Overall, 106 records have been identified, from which 34 records were eligible for analysis (Figure S1). Four studies were considered to be biased (Table S1).


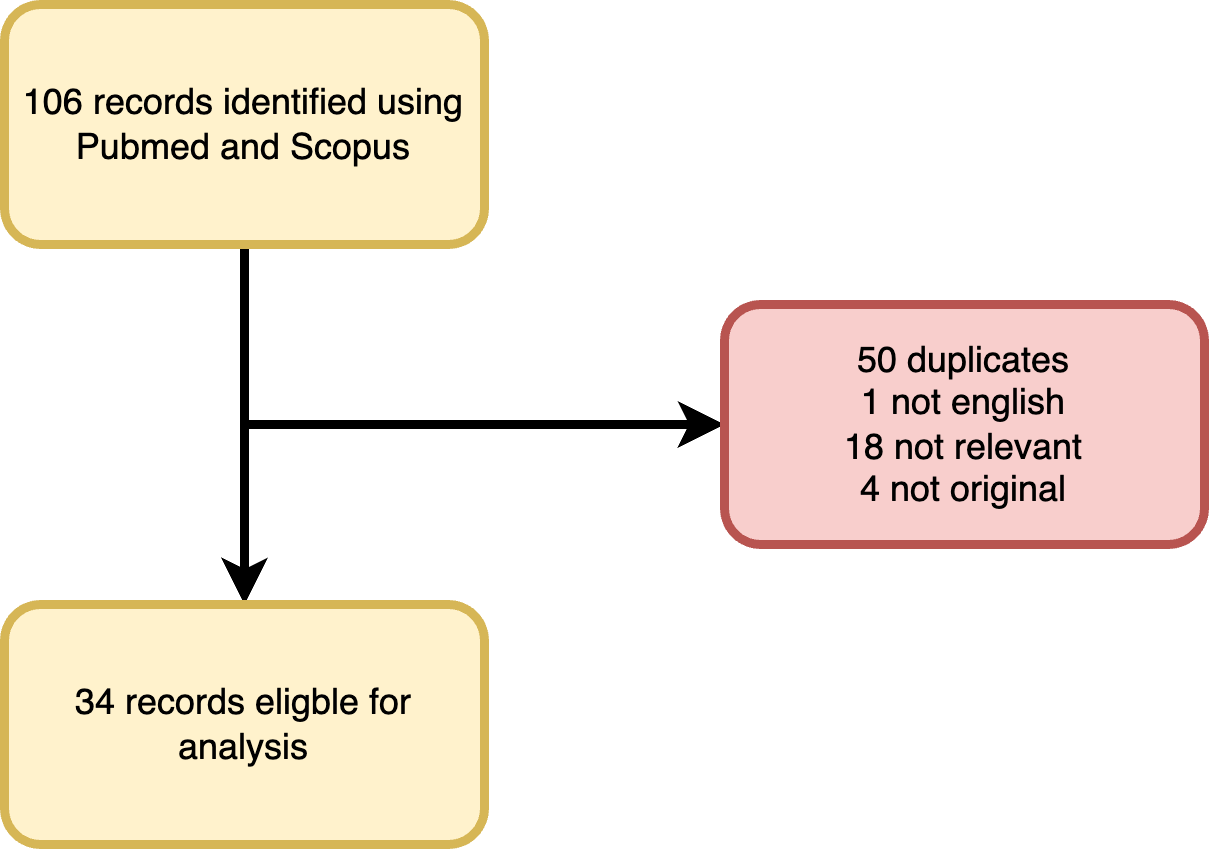


**Figure S1** Flowchart for the review of radiomics papers in 2023 that use resampling methods.

| Author | Title | Reason |
| --- | --- | --- |
| M. Renugadevi, K. Narasimhan, C.V. Ravikumar, R. Anbazhagan, G. Pau, K. Ramkumar, M. Abbas, N. Raju, K. Sathish, P. Sevugan | Machine Learning Empowered Brain Tumor Segmentation and Grading Model for Lifetime Prediction | BraTS2020 has training and testing subsets, with the former including 369 cases with ground truth annotations that identify the tumor parts of the brain scans. Conversely, the testing subset doesn’t have any ground truth annotations and is explicitly used for testing and evaluating the performance of various models and algorithms.  The BraTS2020 MRI images were preprocessed and splitted into 80%, 10% and 10% for training, validation and testing datasets, respectively.  The pseudocode of the SMOTE algorithm is explained  in the Algorithm 1. This is applied to 369 samples in each modality. |
| L. Wang, X. Wu, R. Tian, H. Ma, Z. Jiang, W. Zhao, G. Cui, M. Li, Q. Hu, X. Yu, W. Xu | MRI-based pre-Radiomics and delta-Radiomics models accurately predict the post-treatment response of rectal adenocarcinoma to neoadjuvant chemoradiotherapy. | Multiple group comparison experiments were performed. First, machine learning models were compared using single-model MRI and multi-modal MRI. The T1, T2, and T1+T2 integrated models were then constructed. Second, original features-based models and resampled features-based models using SMOTE were compared. Then, the pre-Radiomics model and delta-Radiomics model were compared through cross-validation with 5-fold and 10-fold, leave-one-out validation, and independent test. |
| S.L. Savaridas, U. Agrawal, A.F. Fagbamigbe, S.L. Tennant, C. McCowan | Radiomic analysis in contrast-enhanced mammography using a multivendor data set: accuracy of models according to segmentation techniques. | Oversampling was performed to balance the class distribution at 2:1 (malignant:benign). Random samples for benign lesions were generated using a verified method, the synthetic minority oversampling technique (SMOTE).23 New samples similar to input samples in the feature space were generated.The data were then split into training and test sets in a ratio of 75:25. |
| M. Szep, R. Pintican, B. Boca, A. Perja, M. Duma, D. Feier, F. Epure, B. Fetica, D. Eniu, A. Roman, S.M. Dudea, A. Chiorean | Whole-Tumor ADC Texture Analysis Is Able to Predict Breast Cancer Receptor Status. | Synthetic Minority Oversampling Technique (SMOTE) was used to improve random oversampling and to augment the 185 patients with 25 more patients. A total of 210 patients (mean age 46.3) were included in the study. The patients were randomly divided into training (150 patients) and validation (60 patients) groups, in a 3:1 ratio |
| B. Dunn, M. Pierobon, Q. Wei | Automated Classification of Lung Cancer Subtypes Using Deep Learning and CT-Scan Based Radiomic Analysis | We used the synthetic minority over-sampling technique (SMOTE) to address the problem of class imbalance in our dataset, in which adenocarcinoma patients (*n* = 251) greatly outnumbered small cell carcinoma patients (*n* = 38) and squamous cell carcinoma patients (*n* = 61).  Table 3. |

**Table S1** List of studies that seemingly misapply the resampling methods together with the text excerpt from which the misapplication can be deduced.


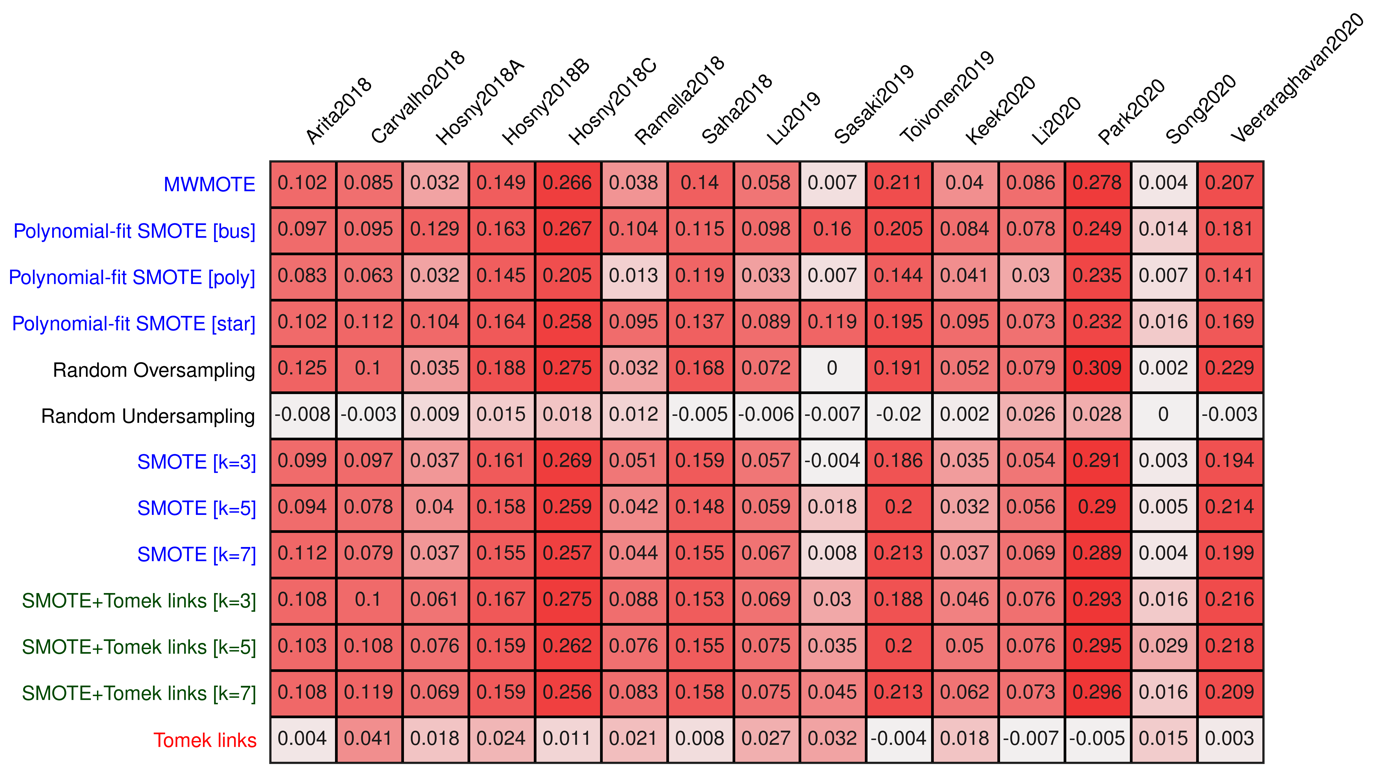


**Figure S2** Bias in sensitivity of the best-performing models averaged over 30 repeats for each method and dataset. Undersampling methods are displayed in red, combined in green, and oversampling methods in red.

**
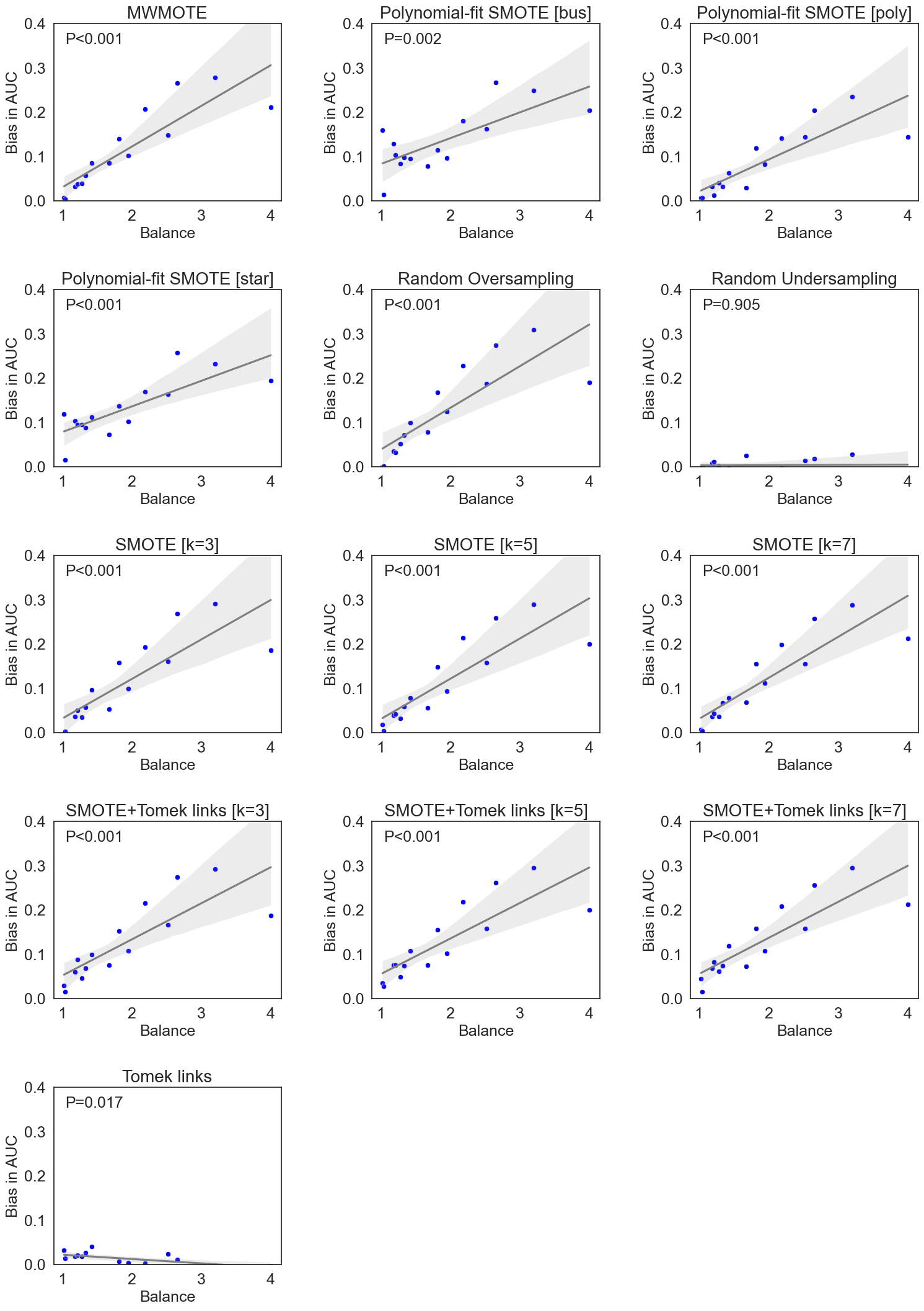
Figure S3** Association of the bias in sensitivity with the class-balance of each dataset. The grey area denotes the 95% confidence interval.


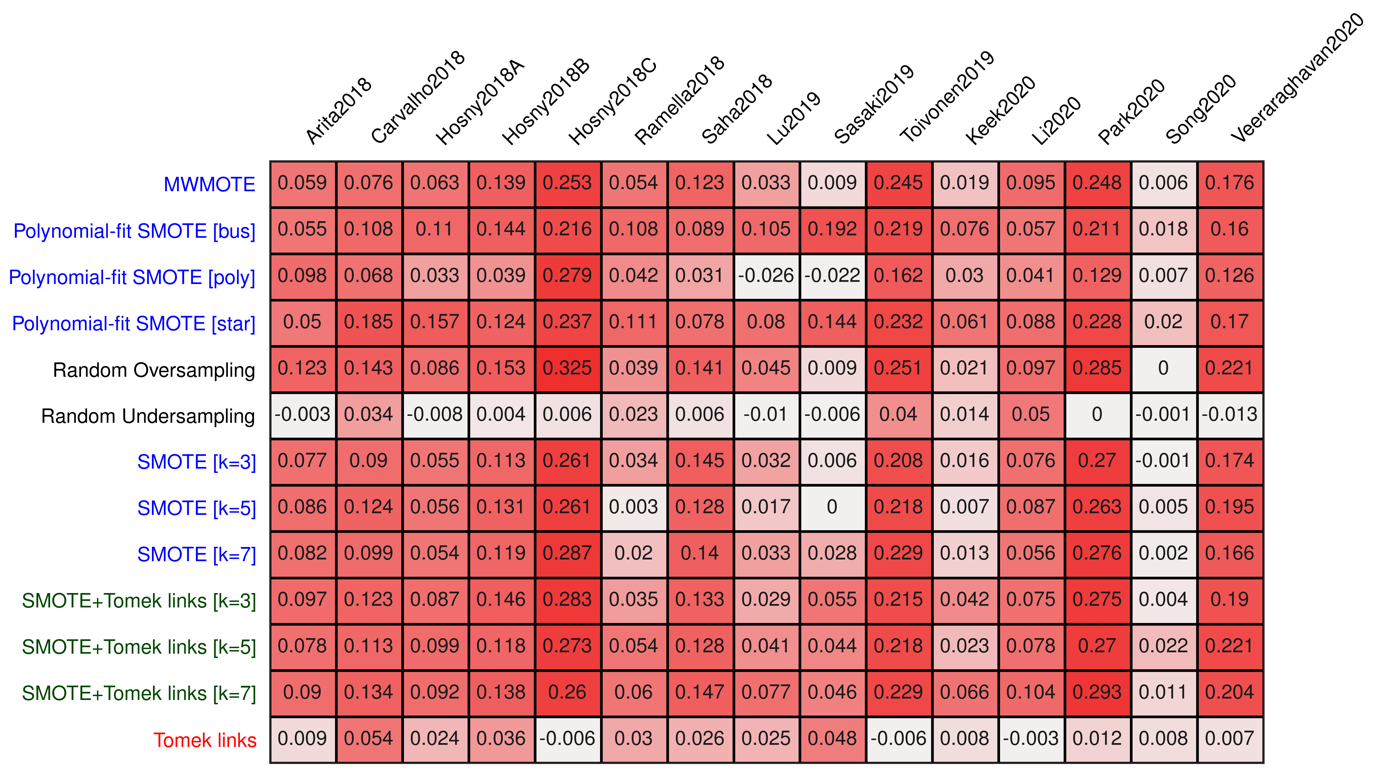


**Figure S4** Bias in specificity of the best-performing models averaged over 30 repeats for each method and dataset. Undersampling methods are displayed in red, combined in green, and oversampling methods in red.

**
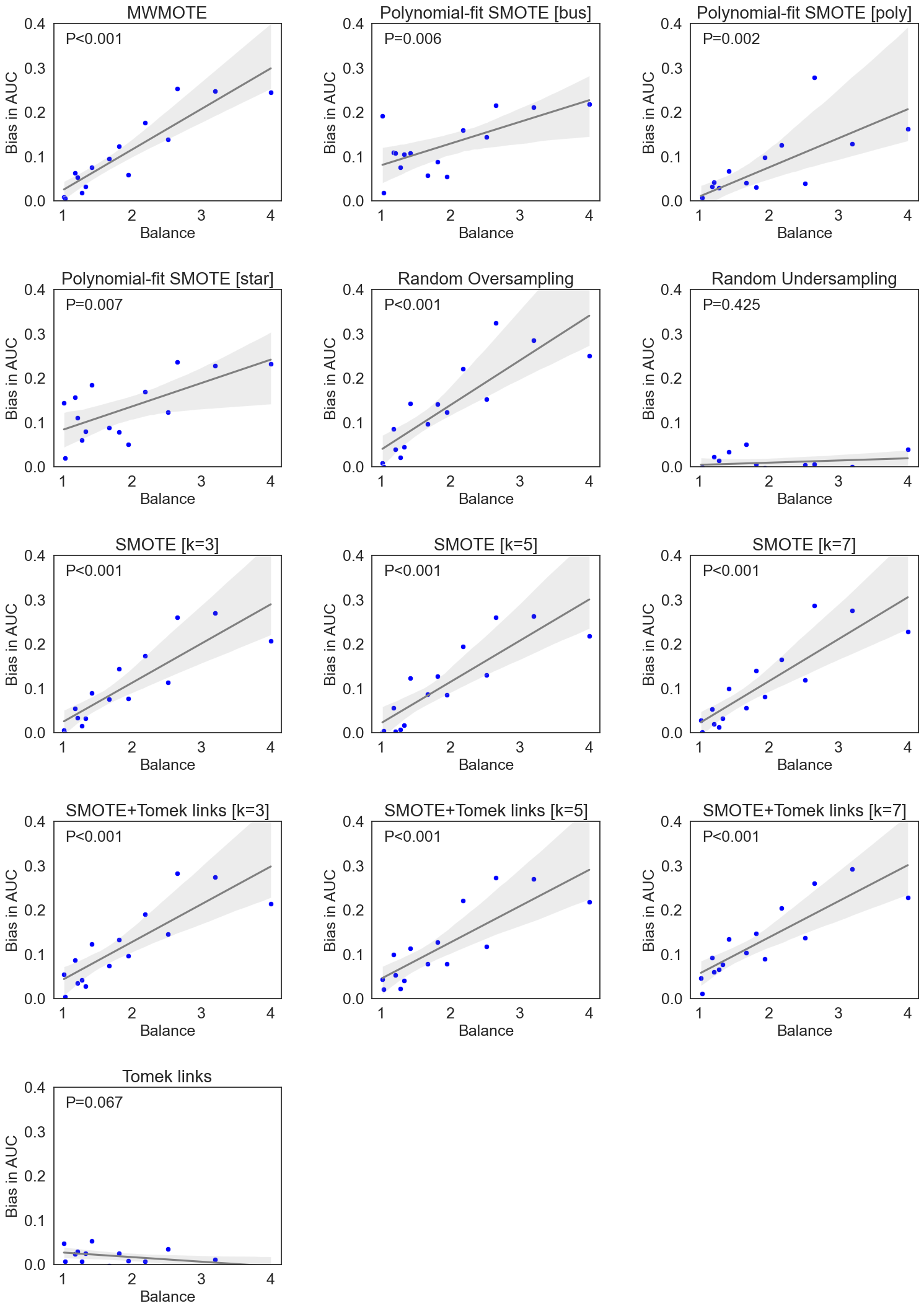
**

**Figure S5** Association of the bias in specificity with the class-balance of each dataset. The grey area denotes the 95% confidence interval.


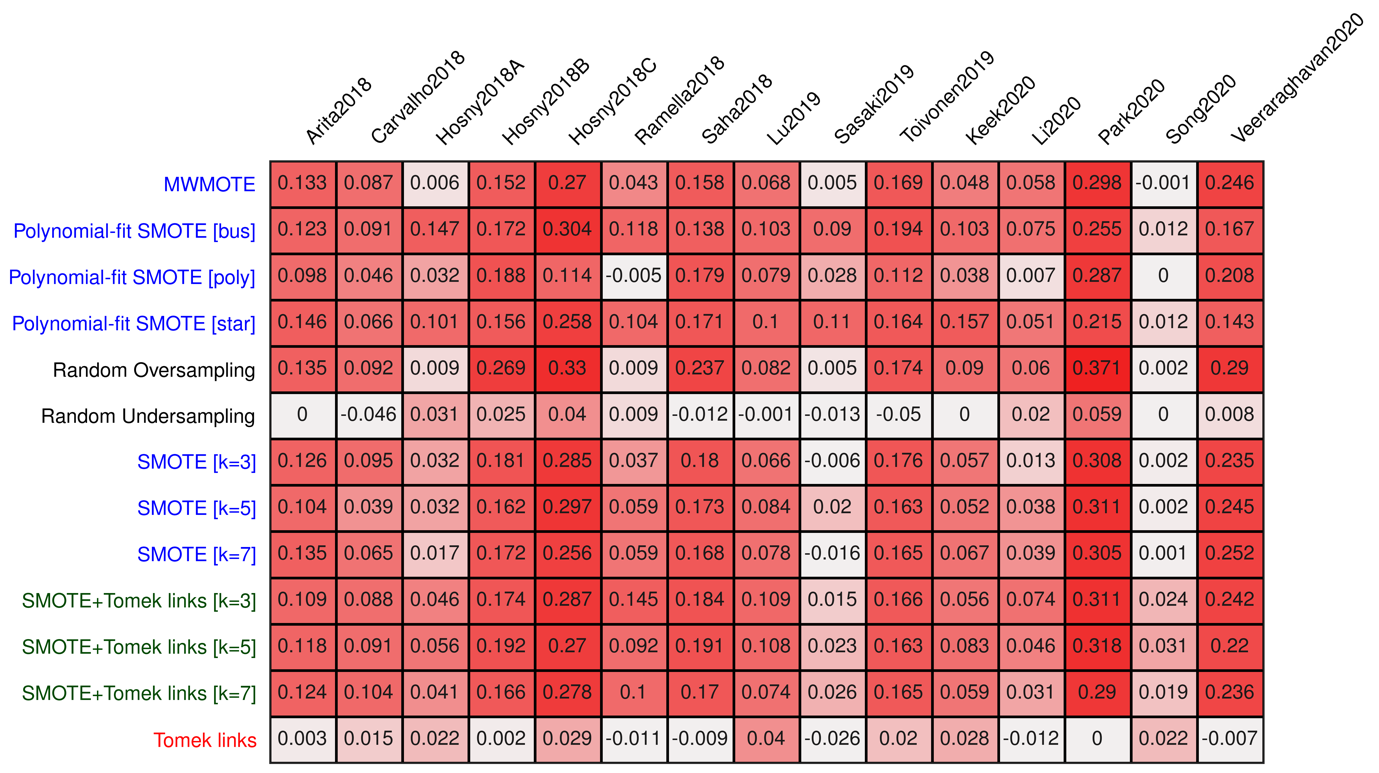


**Figure S6** Bias in balanced accuracy of the best-performing models averaged over 30 repeats for each method and dataset. Undersampling methods are displayed in red, combined in green, and oversampling methods in red.

**
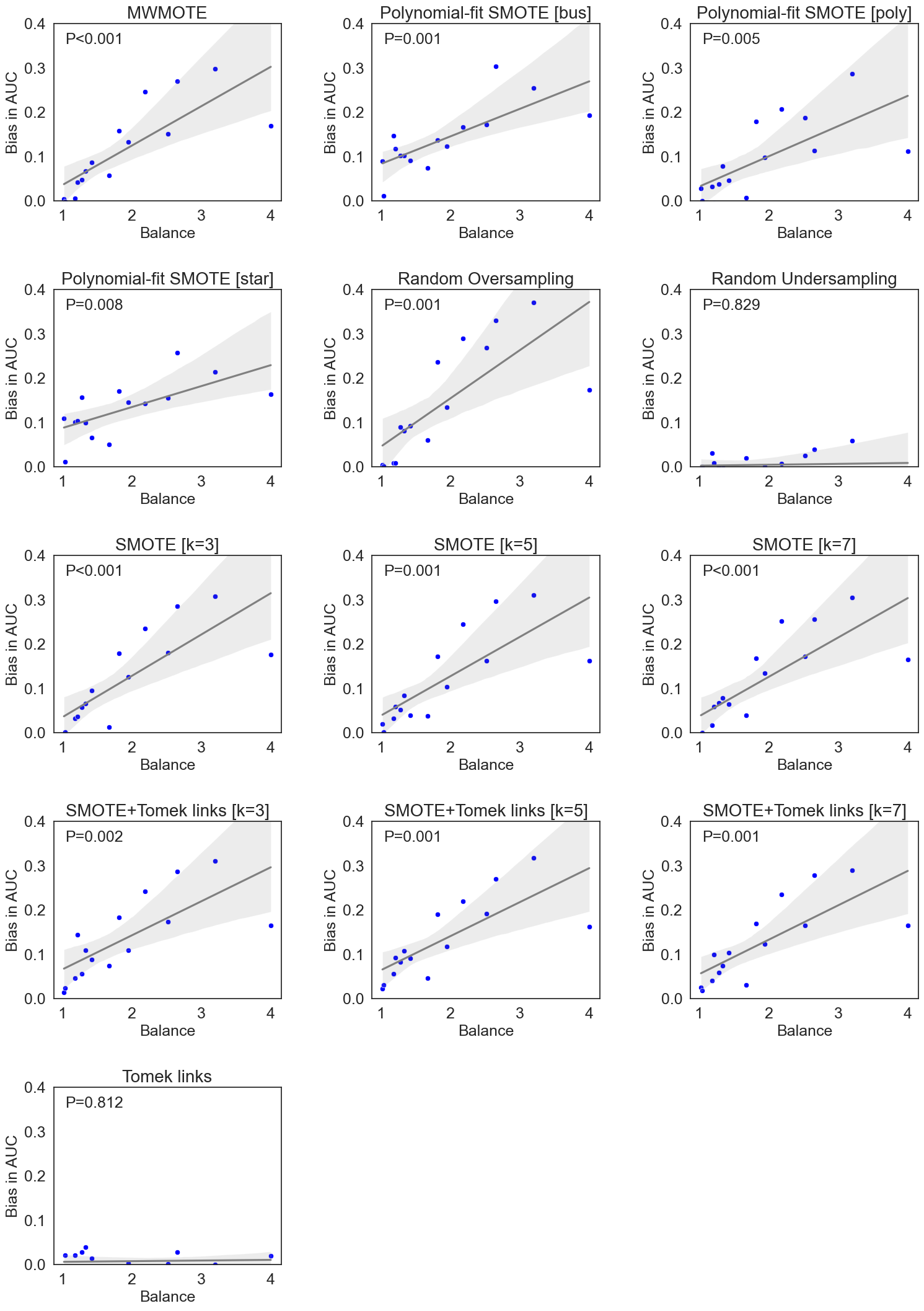
**

**Figure S7** Association of the bias in balanced accuracy with the class-balance of each dataset. The grey area denotes the 95% confidence interval.
